# Supplementary material for: Identify specific gene pairs for subarachnoid hemorrhage based on wavelet analysis and genetic algorithm
Source: PLoS One. 2021 Jun 17;16(6):e0253219. doi: 10.1371/journal.pone.0253219 (PMC8211192; doi:10.1371/journal.pone.0253219)
Supplement: Attachment — Submitted filename: Response to Reviewers.docx [file pone.0253219.s002.docx]

1. The authors should address the format of PLOS ONE to revise the whole paper.

Reply to editor: Thanks for your suggestions. We have revised the manuscript according to the MANUSCRIPT BODY FORMATTING GUIDELINES. The font and text size have been modified accordingly.

2. The research questions should be defined.

Reply to editor: We highlighted this study's aim and the issues we want to address in the abstract section. The primary subjective is to find out new biomarkers for subarachnoid hemorrhage and build a diagnostic predictor.

3. The contributions of this study should be highlight in the first section.

Reply to editor: Thanks for your suggestion. We have included the contributions of this study to the abstract section. The significant contribution is that we developed a new protocol pairing genes and screened significant gene pairs based on the feature selection algorithm. A classifier was constructed with the selected gene pairs and achieved a high performance.

4. A literature review section should be given and discussed.

Reply to editor: We included the literature review in the discussion part. Multiple studies are discussed and verified our signature genes. The conclusion of this study is well concordant to the earlier studies.

5. The authors should recognize the section of the method. They only listed some ideas in the second section. However, they should present the method and how the method can solve the research questions. The quality of the presentation should be improved.

Reply to editor: The method section has been reorganized. We added the description regarding how each method can solve the research questions.

6. The authors should recognize the section of the results. They only listed some results in the third section. However, they should discuss the results and why the method can solve the research questions. The quality of the presentation should be improved.

Reply to editor: The result section is also revised. We added descriptions about why the result contributes to this study.

7. The authors should present the limitation of this study.

Reply to editor: The limitation of this study has been included in the discussion.

8. Future work should be discussed and given in the last section.

Reply to editor: We stated some drawbacks of this study, and even though they are out of the scope of our current study, they will be included in our future work.

Finally, thank you very much for your comments on the language issues of the article, and we have modified it again. However, considering the professionalism and rigor of the language, if you think there are still some problems with the language, I would like to ask if your journal has a recommended service platform for polishing the mother tongue, if so, please tell me the link, thank you.
