# Supplementary material for: Identify specific gene pairs for subarachnoid hemorrhage based on wavelet analysis and genetic algorithm
Source: PLoS One. 2021 Jun 17;16(6):e0253219. doi: 10.1371/journal.pone.0253219 (PMC8211192; doi:10.1371/journal.pone.0253219)
Supplement: Attachment — Submitted filename: Response to Reviewers(new).docx [file pone.0253219.s003.docx]

1. Please review your reference list to ensure that it is complete and correct.

Reply to editor: Thanks for your suggestions. We have checked the reference list and found no retracted papers.

2. Please consider discussing your findings within the context of these two recent publications:

…

Reply to editor: Thanks for your suggestions. We read the above two articles carefully, Xu et al compared the mRNA expression in whole blood of SAH patients with and without vasospasm. The results demonstrate that mRNA expression level signatures can be applied to distinguish SAH patients from normal controls. Another study also evaluated the relationship between aSAH patient outcomes and genetic variants and DNA methylation. The author found that DNA methylation of hepcidin geneplayscritical roles in patients following aSAH. Both studies imply the potential application of gene signatures as diagnostic and prognostic biomarkers for aSAH patients. We have added the above in the introduction(Manuscript Page2) and have added references from both articles to our list of references(Manuscript Page13).
